# Postural asymmetries in young adults with cerebral palsy

**DOI:** 10.1111/dmcn.12199

**Published:** 2013-07-09

**Authors:** Elisabet Rodby-Bousquet, Tomasz Czuba, Gunnar Hägglund, Lena Westbom

**Affiliations:** 1 Centre for Clinical Research, Uppsala University, Central HospitalVästerås, Sweden; 2 Department of Orthopaedics, Lund University, Skåne University HospitalLund, Sweden; 3 RC Syd, National Centre for Quality Registers, Lund University, Skåne University HospitalLund, Sweden; 4 Department of Paediatrics, Lund University, Skåne University HospitalLund, Sweden

## Abstract

**Aim** The purpose was to describe posture, ability to change position, and association between posture and contractures, hip dislocation, scoliosis, and pain in young adults with cerebral palsy (CP).

**Methods** Cross-sectional data of 102 people (63 males, 39 females; age range 19–23y, median 21y) out of a total population with CP was analysed in relation to Gross Motor Function Classification System (GMFCS) levels I (*n*=38), II (*n*=21), III (*n*=13), IV (*n*=10), and V (*n*=20). The CP subtypes were unilateral spastic (*n*=26), bilateral spastic (*n*=45), ataxic (*n*=12), and dyskinetic CP (*n*=19). The Postural Ability Scale was used to assess posture. The relationship between posture and joint range of motion, hip dislocation, scoliosis, and pain was analysed using logistic regression and Spearman’s correlation.

**Results** At GMFCS levels I to II, head and trunk asymmetries were most common; at GMFCS levels III to V postural asymmetries varied with position. The odds ratios (OR) for severe postural asymmetries were significantly higher for those with scoliosis (OR=33 sitting), limited hip extension (OR=39 supine), or limited knee extension (OR=37 standing). Postural asymmetries correlated to hip dislocations: supine (*r*^s^=0.48), sitting (*r*^s^=0.40), standing (*r*^s^=0.41), and inability to change position: supine (*r*^s^=0.60), sitting (*r*^s^=0.73), and standing (*r*^s^=0.64).

**Conclusions** Postural asymmetries were associated with scoliosis, hip dislocations, hip and knee contractures, and inability to change position.

This article is commented on by Novak on page 974 of this issue.

Disorders of posture and movement are key problems in cerebral palsy (CP),^1^ with half the population requiring assistance to stand or walk because of difficulties with aligning and stabilizing themselves against gravity.

An asymmetric posture increases the risk of tissue adaptation, leading to contractures and progressive deformities.^2^–^4^ Contractures, and bone and joint deformities most commonly affect the lower extremities and the spine, leading to scoliosis, pelvic obliquity, hip dislocations, windswept deformities, flexed hips and knees, and foot deformities.^5^ To increase function and minimize the risk of musculoskeletal deformities there is sometimes a need to align and stabilize the body segments and reduce the impact of gravity by providing appropriate support. In children with CP, approximately 30% to 40% use assistive devices to stand or sit.^6^ People with CP who are non-ambulant are more vulnerable to development of contractures and deformities.^7^,^8^ It is the amount of time spent in an abnormal posture that is critical to the development of a contracture. The longer a posture is held, the greater the risk for contracture.^9^

There is a decline in gross motor function in adults with CP such as reduced balance, joint range of motion (ROM), and increased pain.^10^–^12^ Scoliosis curve magnitude tends to increase with age even after bone maturity.^7^ However, contractures, hip dislocations, scoliosis, and other fixed deformities can be reduced by early detection and preventive treatment.^13^–^16^ In Sweden a national health care program and quality register for children with CP (CPUP) was started in 1994 as an attempt to prevent hip dislocations and contractures.^13^,^14^,^16^ CPUP includes children and adolescents with CP until 18 years of age. CP is a lifelong condition and therefore a project was started in 2009 to expand CPUP to also include adults. An assessment form was developed for the examinations (http://www.cpup.se), including clinical assessment of passive ROM, spine, posture, mobility, gross motor function, and information about pain, etc.

The purpose of this study was to describe posture in supine, sitting, and standing positions, the ability to change position, and also to analyse the association between posture and limited ROM, hip dislocation, scoliosis, and pain in young adults with CP.

## Method

A descriptive cross-sectional study of a total population was performed, including 102 adults with CP (63 males, 39 females) born between 1988 and 1991, based on data from the CPUP health care program for adults in the south of Sweden. Data included all people examined from the start of October 2009 until the end of December 2011. The study was approved by the Medical Research Ethics Committee at Lund University, LU 443-99, and informed consent was given by the participants.

### Population and participants

The study population comprised adults with CP born between 1988 and 1991, living in the two southernmost counties of Sweden (Skåne and Blekinge) on the 1st of January 2009. People born between 1990 and 1991 were previously followed by the CPUP program for children, but before the hip screening started. Adults with CP born between 1988 and 1989 were usually followed by the child rehabilitation units until 20 years of age, and continued rehabilitation was offered at rehabilitation units for adults. Adolescents with mild functional impairments were usually not followed by the rehabilitation services after adolescence. The reports in the CPUP register are based on regular neuropaediatric inventories of medical records and diagnosis lists from hospitals and rehabilitation units. The CP diagnosis and subtypes were validated by a senior neuropaediatrician during the inventories.^17^ Adults born between 1988 and 1989 were included in the inventory for 2009.

**Figure 1:**
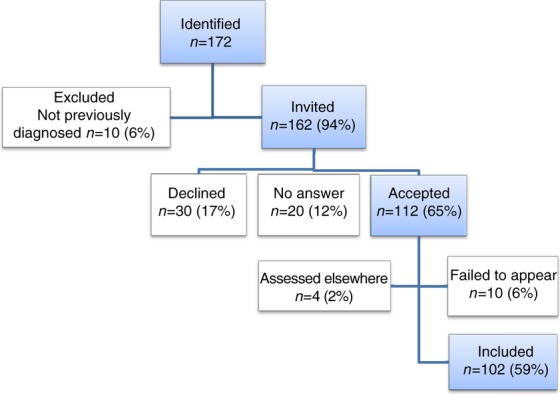
The number of adults with cerebral palsy being identified, invited, and included in the study, and the number of individuals excluded and the drop-outs.

Place of residence on the 1st of January 2009 was ascertained through the Swedish population register (Statistics Sweden).^18^ A total of 172 people with CP born between 1988 and 1991 were living in the area at that date; corresponding CP prevalence was 2.3 per 1000 at age 17 to 20 years. In ten people born between 1988 and 1989 CP was unrecognized in the medical records; they were probably not informed about the diagnosis and were not approached. Invitations to the CP follow-up program were sent to 162 people, of which 26 declined, 20 did not answer, and 116 accepted. Four of them were recently assessed in the child rehabilitation services according to their CPUP program, and ten failed to appear before the end of 2011 (Fig. 1). The remaining 102 people (63 males, 39 females) took part in this study at 19 to 23 years of age (mean 20y 6mo). There were no statistically significant differences found between the characteristics of participants and non-participants, except the proportion of unknown levels of gross motor function (Table 1).

**Table 1:** The total population of adults with cerebral palsy (CP) born between 1988 and 1991 living in Skåne and Blekinge on the 1st of January 2009, study participants, and non-participants

	Total population *n*=172	Participants *n*=102	Non-participants *n*=70
Region			
Skåne	154 (89.5%)	91 (89%)	63
Blekinge	18 (10.5%)	11 (11%)	7
Sex			
Female	68 (39.5%)	39 (38%)	29
Male	104 (60.5%)	63 (62%)	41
Birth year			
1988	41 (24%)	21 (21%)	21
1989	41 (24%)	29 (28%)	12
1990	41 (24%)	24 (24%)	17
1991	49 (28%)	28 (27%)	21
Subtype			
USCP	46 (27%)	26 (25%)	20
BSCP	72 (42%)	45 (44%)	27
Dyskinetic CP	28 (16%)	19 (19%)	9
Ataxic CP	25 (14.5%)	12 (12%)	13
Unclassified	1 (0.5%)	0 (0%)	1
GMFCS level			
I	63 (36.5%)	38 (37%)	26
II	24 (14%)	21 (21%)	3
III	19 (11%)	13 (13%)	6
IV	14 (8%)	10 (10%)	4
V	26 (15%)	20 (20%)	6
Unknown	25 (14.5%)	0 (0%)	25

USCP, unilateral spastic cerebral palsy; BSCP, bilateral spastic cerebral palsy; GMFCS, Gross Motor Function Classification System.

### Classifications and measurements

Cerebral palsy was defined according to Rosenbaum et al.^1^ The CP diagnosis was confirmed and the neurological subtypes were classified by a neuropaediatrician according to the Surveillance of Cerebral Palsy in Europe network (SCPE),^19^ with the non-progressive brain dysfunction arising before the age of two. The CP subtypes were classified into unilateral spastic (USCP), bilateral spastic (BSCP), ataxic, and dyskinetic CP (Table 1).

**What this paper adds**

- Postural asymmetries are present at all GMFCS levels in young adults with CP.
- Postural asymmetries are associated with contractures, deformities, and inability to change position.
- Fifty percent of adults at GMFCS levels IV and V have only one sleeping position.

All examinations were made by physiotherapists and occupational therapists according to an assessment form and an accompanying manual (http://www.cpup.se/se/index.php/tack.html). Gross motor function was determined according to the expanded and revised version of the Gross Motor Function Classification System (GMFCS), which comprises five levels (I–V).^20^ Level V represents the most severe functional limitations; for distribution see Table 1. The age band 12 to 18 years was used. Posture was assessed using items from the first version of the Posture and Postural Ability Scale^21^ called the Postural Ability Scale (PAS)^22^ from the early 1990s. It has shown excellent interrater reliability, high internal consistency, and construct validity for adults with CP at all GMFCS levels.^21^ Any deviations from midline in head, trunk, leg, foot position, or asymmetries in arm position or weight bearing gives 0 point each and symmetric, neutral position gives 1 point each with a total score of 0 to 6 points where a maximum score of 6 points indicates full symmetry (for items see Table 2). When a person cannot be placed in a position due to severe contractures, posture is scored as 0 points.

**Table 2:** Distribution of postural asymmetries according to the Postural Ability Scale (PAS) in supine, sitting, and standing position for each Gross Motor Function Classification System (GMFCS) level I–V

Position	PAS items	I *n*=38	II *n*=21	III *n*=13	IV *n*=10	V *n*=20
		*n* (f)	*n* (f)	*n* (f)	*n* (f)	*n* (f)
Supine	Head midline*	3 (0.1)	7 (0.4)	1 (0.1)	2 (0.2)	11 (0.6)
	Trunk symmetric*	3 (0.1)	7 (0.4)	3 (0.3)	2 (0.2)	15 (0.8)
	Legs straight relative to pelvis*	2 (0.1)	6 (0.3)	7 (0.6)	7 (0.7)	19 (1.0)
	Legs separated*	0 (0)	2 (0.1)	4 (0.3)	6 (0.7)	12 (0.6)
	Arms resting by side*	3 (0.1)	3 (0.2)	1 (0.1)	7 (0.7)	18 (1.0)
	Weight evenly distributed*	0 (0)	4 (0.2)	5 (0.4)	7 (0.7)	17 (0.9)
Sitting	Head midline*	1 (0)	5 (0.3)	2 (0.2)	3 (0.3)	11 (0.7)
	Trunk symmetric*	4 (0.1)	8 (0.4)	5 (0.4)	6 (0.6)	12 (0.7)
	Legs separated and in neutral position*	1 (0)	2 (0.1)	2 (0.1)	5 (0.5)	12 (0.7)
	Arms resting by side*	2 (0.1)	1 (0.1)	2 (0.2)	6 (0.6)	16 (0.9)
	Both feet flat on floor*	0 (0)	1 (0.1)	1 (0.2)	5 (0.5)	12 (0.7)
	Weight evenly distributed*	2 (0.1)	3 (0.2)	3 (0.2)	6 (0.7)	14 (0.8)
Standing	Head midline	7 (0.2)	7 (0.4)	3 (0.3)	2 (0.3)	3 (0.4)
	Trunk symmetric*	9 (0.3)	14 (0.8)	5 (0.5)	3 (0.6)	4 (0.6)
	Legs straight hips and knees extended*	4 (0.1)	7 (0.4)	8 (0.7)	5 (0.8)	5 (0.7)
	Legs separated*	0 (0)	2 (0.1)	5 (0.5)	4 (0.7)	2 (0.3)
	Both feet flat on floor*	2 (0.1)	1 (0.1)	4 (0.4)	3 (0.5)	4 (0.5)
	Weight evenly distributed	9 (0.3)	11 (0.6)	5 (0.5)	4 (0.7)	4 (0.6)

Results presented as number of individuals and fractions (f) who scored 0 = No, at each item. Fisher’s exact test showed significant differences (*p*<0.01) between GMFCS levels for all items marked.

* *P *> 0.05 for remaining items.

Passive ROM was assessed by goniometric measurement and classified as limited if extension of hips, knees, or elbows were less than 0 degrees on one or both sides or inability to reach 0 degrees of dorsiflexion of the feet (Table 3). Scoliosis was defined as either having a spinal curve at clinical examination or had been operated with a spinal fusion due to scoliosis. Hip dislocation was determined by an orthopaedic surgeon from radiographs and defined as Reimers’s migration percentage of 100% in at least one hip.^23^ Pain was reported either by client or by proxy as yes or no to any presence of pain during the last 4 weeks (Table 3). The use of standing supports were reported by client or by proxy as more than 1, 1 to 2, 2 to 3, or 3 to 4 hours per day. The ability to maintain and change position (independently, with support, cannot/or/need total assistance) and the sleeping positions (side lying right/left, prone, supine) were reported by client or by proxy.

**Table 3:** Presence of hip dislocation, scoliosis, limited range of motion, and pain in relation to Gross Motor Function Classification System level

	I *n*=38	II *n*=21	III *n*=13	IV *n*=10	V *n*=20	Total *n*=102
	*n* (f)	*n* (f)	*n* (f)	*n* (f)	*n* (f)	*n* (f)
Hip dislocation	0 (0)	0 (0)	1 (0.1)	1 (0.1)	8 (0.4)	10 (0.1)
Scoliosis	12 (0.4)	7 (0.3)	6 (0.5)	6 (0.6)	17 (0.9)	48 (0.5)
Limited elbow extension	11 (0.3)	6 (0.3)	5 (0.4)	6 (0.6)	10 (0.6)	38 (0.4)
Limited hip extension	0 (0)	3 (0.2)	4 (0.3)	3 (0.3)	11 (0.6)	21 (0.2)
Limited knee extension	18 (0.5)	6 (0.3)	10 (0.8)	8 (0.8)	18 (0.9)	60 (0.6)
Limited foot dorsiflexion	7 (0.2)	8 (0.4)	3 (0.2)	3 (0.3)	4 (0.2)	25 (0.3)
Pain	23 (0.6)	13 (0.6)	7 (0.5)	8 (0.8)	12 (0.6)	63 (0.6)

Results presented as number of individuals and fractions (f).

### Statistical analyses

Z-test comparison of column proportions after Bonferroni adjusted *p*-values was used to analyse differences between participants and non-participants. Spearman’s correlation test, with 95% non-parametric bootstrap confidence intervals (CI) based on 1000 re-samples, was used to analyse the relationship between asymmetric postures and categorical variables such as hip dislocation (yes or no). Fisher’s exact test and Spearman’s correlation test were used to analyse the relationships between ordinal variables such as GMFCS level I to V and PAS total score 0 to 6 points. Logistic regression was used to model the relationship between asymmetric posture and limited ROM, hip dislocation, scoliosis, and pain. In this case postural asymmetries were treated as no asymmetry (6 points), mild (5–4 points), moderate (3–2 points), and severe (1–0 points). The results were presented as odds ratios (OR). If the odds are identical in each group, the odds ratio is equal to one. R software environment (R Foundation, Vienna, Austria) and stata 12 (Statistics/Data Analysis, StataCorp, College Station, TX, USA) were used for statistical analyses.

## Results

### Posture, range of motion, and pain

Postures were present at all GMFCS levels but more frequently at lower levels of gross motor function. The GMFCS correlated to postural asymmetries in supine *r*^s^=−0.78 (CI −0.84 to −0.65), sitting *r*^s^=−0.75 (CI −0.83 to −0.61), and standing *r*^s^=−0.69 (CI −0.81 to −0.53). There was also a correlation between postures, where lying posture correlated to posture in sitting *r*^s^=0.78 (CI 0.65–0.86) and standing *r*^s^=0.76 (CI 0.63–0.84).

At GMFCS level I to II head and trunk asymmetries were more common while asymmetries varied more due to position at GMFCS level III to V (Table 2). At GMFCS level III a higher proportion did not have legs separated in supine and standing which requires extended legs, compared to sitting where hips and knees are flexed. There were more postural asymmetries in standing compared to supine lying and sitting for individuals at GMFCS level I to III. The reverse was seen at GMFCS level V with less asymmetry in standing with support compared to supine lying and sitting (Table 2).

Some limitations of knee, foot, and elbow extension were present at all GMFCS levels while limited hip extension was found at GMFCS level II to V and dislocated hips at GMFCS level III to V (Table 3). Pain was reported by 63 of 102 individuals (Table 3). No significant correlation was found between posture and pain. Hip dislocations showed a fair correlation to postural asymmetries in supine *r*^s^=0.48 (CI 0.34–0.63), in sitting *r*^s^=0.40 (CI 0.28–0.57), and in standing *r*^s^=0.41 (CI 0.25–0.55). Inability to put feet flat on floor in standing correlated to limited knee extension *r*^s^=0.40 (CI 0.22–0.54) but not to limited dorsiflexion of the feet.

The odds ratios for severe postural asymmetries were significantly higher for those with scoliosis (OR=33 in sitting), limited hip extension (OR=39 in supine), or limited knee extension (OR=37 in standing; Table 4). Also, limited elbow extension increased the odds ratio for postural asymmetries. Pain, as measured in this study, did not influence the odds for asymmetric posture.

**Table 4:** Odds ratio (OR) with 95% confidence interval (CI) for postural asymmetry

Position	Explanatory variable	5–4 points	CI	3–2 points	CI	1–0 points	CI
Supine	Limited knee extension	2	0.7–6.7	10	2.5–38	11	2.1–53
	Limited hip extension	2	0.3–18	13	2.4–70	39	6.1–250
	Limited elbow extension	2	0.6–6.9	6	1.7–18	4	1.2–16
	Scoliosis	2	0.7–6.2	3	1.1–8.9	26	3.1–218
Sitting	Limited elbow extension	4	1.2–11	5	1.3–20	5	1.5–20
	Scoliosis	3	1.0–7.6	5	1.3–19	33	4–281
Standing	Limited knee extension	2	0.6–6.8	13	2.7–62	37	6.8–207
	Limited hip extension	8	0.8–70	8	0.8–76	18	2–154
	Limited elbow extension	5	1.0–20	6	1.3–32	11	2.6–49
	Scoliosis	2	0.7–7.3	2	0.6–7.7	9	2.6–34

Postural asymmetry according to the Postural Ability Scale was divided into mild (5–4 points), moderate (3–2 points), and severe asymmetry (1–0 points). Full symmetry (6 points) was used as reference category.

### Maintain and change position

Inability to change position correlated to postural asymmetries in supine *r*^s^=0.60 (CI 0.47–0.73), sitting *r*^s^=0.73 (CI 0.60–0.82), and in standing *r*^s^=0.64 (CI 0.45–0.76).

All adults at GMFCS level I to III maintained lying position independently, while 10% of those at GMFCS level IV and 60% at GMFCS level V needed support. The correlation between GMFCS and ability to change position in lying was *r*^s^=0.67 (CI 0.54–0.76). Fifty percent of the adults at GMFCS levels IV and V had only one lying position, the other half changed between two or three positions. Only eight adults, all at GMFCS level I to III, changed between all four positions prone, supine, and side lying left and right. All 47 individuals with a total score of 5 to 6 points (full or almost full symmetry) for supine posture changed position independently in lying. Seven out of nine with a total score of 0 points (total asymmetry) could not change position and required total assistance; six of them had only one sleeping position.

At GMFCS levels IV and V everyone used postural support to maintain sitting. The ability to change position from sit to stand showed a high correlation to GMFCS levels *r*^s^=0.88 (CI 0.83–0.93), where 86% at GMFCS level II and 31% at GMFCS level III moved from sit to stand without any support. Support was used by 69% at GMFCS level IV and 60% at GMFCS level V. The remaining 40% at GMFCS level V could not move from sit to stand even with support. Of the 42 individuals with a total score of 6 points, 37 (88%) moved from sit to stand without any support while nine of 10 with a total score of 0 points in sitting could not move from sit to stand even with support.

All individuals at GMFCS level I to II and 38% at level III stood unsupported, while supported standing was used by 46% at level III, 90% at level IV, and 74% at GMFCS level V. The remaining 26% at GMFCS level V did not stand at all. The correlation of standing ability to GMFCS was *r*^s^=0.69 (CI 0.46–0.83). Of the 26 persons using standing support; seven stood 1 to 2 hours per day and the remaining 19 stood less than 1 hour per day. A fully symmetric standing posture was more frequent in those who maintained standing independently. Of the 25 people with a total score of 6 points 23 (92%) stood without support, while none of the four individuals with an asymmetric posture and a score of 0 points did. Of the 25 people with a fully symmetric standing posture 22 (88%) could change position and move from stand to sit independently, while all four with a totally asymmetric standing posture needed support or could not move from stand to sit even with support.

## Discussion

This is, to our knowledge, the first study of postural asymmetries in a total population of adults with CP.

Postural asymmetries were present in adults at all GMFCS level, but more frequent at lower levels of motor function and varied in different positions. Normally a standing position requires more postural ability, and those at GMFCS level I to III demonstrated more asymmetries in standing compared to sitting and supine lying. However the reverse was seen at GMFCS level V with a higher proportion of postural asymmetries in supine and sitting compared to supported standing, indicating a lack of postural support while lying and sitting.

The time spent in different positions may have a great impact on the development of contractures and deformities. In this study no one who used standing support stood more than 1 to 2 hours per day. This implies that 22 to 24 out of the 24 hours per day were spent in a more asymmetric position in sitting or lying for those at GMFCS level V. In addition they could not change their position while lying or sitting. Of those who were unable to change position in lying half had only one lying position, indicating that they were not assisted in changing position. Porter et al.^4^,^8^ showed that preferred lying postures influence the direction of deformity with windsweeping, hip dislocation, and spinal curve in children with CP unable to move out of their preferred posture. A study by Pountney et al.^15^ on posture management to prevent hip dislocation supports the importance of maintaining symmetry without compromising function for those unable to change position. This highlights the need for a proper assessment of posture, and provision of postural support when needed, to prevent a sustained asymmetric posture.

Pain was reported by 63 of the 102 participants but no significant association between posture and pain was found in this study. There was less reported pain compared to previous studies of adults with CP by Jahnsen et al.^11^ (82%) and Andersson and Mattsson^12^ (79%). It may be due to the older age of participants in their studies (mean age 34y and 36y respectively). Another reason could be that pain may be unrecognized in some of the participants in the present study, as people with severe intellectual and communication disabilities were included.

Limited hip and knee extension were highly associated with postural asymmetries. Andersson and Mattson^12^ reported contractures in 80% of 221 adults with CP; knee contractures were most frequent. In the present study knee contractures were also most common; 60 of the 102 adults with CP could not passively extend one or both knees to 0 degrees. Limited hip and knee extension were associated with postural asymmetries in both supine and standing positions which require extended legs.

Previous studies^2^–^4^ indicate that a sustained asymmetric posture may cause progressive deformities in people with CP. This study showed an association between posture and limited ROM but did not reveal if the contractures were caused by asymmetric posture or if the limited ROM caused the postural asymmetries. However, this illustrates the importance of continuous monitoring of ROM and posture in people with CP, to allow early identification and preventive treatment to maintain ROM and symmetric posture.

Scoliosis and hip dislocations were associated with postural asymmetries in all three positions. The prevalence of hip dislocations (10/102) in this material, not included in the hip prevention program, corresponds to reports from other areas.^24^ Hip dislocation, windswept-deformity, and scoliosis are interrelated^16^ and can be reduced with a hip surveillance program. Progression of scoliosis increases with age even after skeletal maturity. Risk factors are early onset, large curve magnitude, thoracolumbar curve, total body involvement, and being confined to bed.^7^ Since 1995 all children in the study area born 1992 and later are included in a hip surveillance program, which have reduced the proportions of hip dislocations, windswept deformities, and scoliosis.^14^,^16^ The association between these deformities and postural asymmetries shows the value of hip surveillance programs.

A limitation of this study is the lack of radiographs of the spinal curves defined as scoliosis. Structural scoliosis are rare at GMFCS level I and flexible spinal curves of postural origin are found to be more frequently rated as scoliosis at clinical examination than at radiographs.^25^ Since no radiographs of the spine were available, we chose to include all spinal curves rated as mild, moderate, or severe by the local physiotherapist and individuals operated with spinal fusion. This is likely to give a higher frequency of scoliosis compared to other studies with different definitions of scoliosis.

Another limitation is the restricted number of participants when analyzing the results for each GMFCS level separately. Although the proportion of participants may seem low (63% of the 162 invited), it is compensated by the total population approach, which is a strength of the study. According to the drop-out analysis the study group is a representative part of the total population, with the CP prevalence 2.3 per 1000 at 17 to 20 years of age. Part of the study population was included in the cohort born 1990 to 1993 with CP prevalence 2.4 per 1000 at 4 to 7 years and 2.8 per 1000 at 8 to 11 years of age.^17^ The distribution of subtypes in the present study almost equals that of the previous studies. According to the prevalence and distribution of sex, subtypes, and GMFCS levels, the study population is likely to be representative for other areas and countries with similar development.^17^

This study illustrates the importance of monitoring ROM and posture from an early age, but also continuously in adults with CP, to allow early identification and preventive treatment of contractures and postural asymmetries.

## Abbreviations

CPUP: Cerebral palsy follow-up program and quality register
PAS: Postural Ability Scale
ROM: Joint range of motion
